# Stage duration distributions in matrix population models

**DOI:** 10.1002/ece3.4279

**Published:** 2018-07-16

**Authors:** Toshinori Okuyama

**Affiliations:** ^1^ Department of Entomology National Taiwan University Taipei Taiwan

**Keywords:** individual variation, maximum likelihood, mixture distributions, stage duration, stage‐structured models

## Abstract

Matrix population models are a standard tool for studying stage‐structured populations, but they are not flexible in describing stage duration distributions. This study describes a method for modeling various such distributions in matrix models. The method uses a mixture of two negative binomial distributions (parametrized using a maximum likelihood method) to approximate a target (true) distribution. To examine the performance of the method, populations consisting of two life stages (juvenile and adult) were considered. The juvenile duration distribution followed a gamma distribution, lognormal distribution, or zero‐truncated (over‐dispersed) Poisson distribution, each of which represents a target distribution to be approximated by a mixture distribution. The true population growth rate based on a target distribution was obtained using an individual‐based model, and the extent to which matrix models can approximate the target dynamics was examined. The results show that the method generally works well for the examined target distributions, but is prone to biased predictions under some conditions. In addition, the method works uniformly better than an existing method whose performance was also examined for comparison. Other details regarding parameter estimation and model development are also discussed.

## 1 INTRODUCTION

The growth rate of a population is determined by the survival and reproduction of its individuals. To understand how individual‐level demographic processes translate to population growth, variation in demographic rates among individuals must be considered. Although trivial, only reproductively mature individuals contribute directly to immediate population growth. Survival rates also vary by life stages (Pinder, Wiener, & Smith, 1978). For example, in the loggerhead sea turtle *Caretta caretta*, the annual survival rate varies among life stages: eggs, juveniles, and adults (Crouse, Crowder, & Caswell, 1987). Ignoring the stage structure of a population can lead to misleading predictions of population growth. Matrix population models are one of the commonly used tools to build stage‐structured models that account for variation in demographic rates among life stages (Caswell, 2001). By explicitly considering distinct life stages, matrix models can identify key demographic parameters that influence population growth, which is highly valuable in applied fields and others (e.g., Crouse et al., 1987; Parker, 2000; Shyu & Caswell, 2016). To establish the relationship between population growth and demographic parameters, proper identification and characterization of life stages are essential.

Unless a stage is defined by a fixed duration (e.g., age), stage duration will vary among individuals within the stage. For example, in an egg stage, some eggs hatch (e.g., become larvae in some insects) before other eggs, even when they were laid at the same time (e.g., Fang, Okuyama, Wu, Feng, & Hsu, 2011; King, Brewer, & Martin, 1975). The distribution of stage duration is another important detail that affects population growth (de Valpine, Scranton, Knape, Ram, & Mills, 2014). However, in matrix models, it is uncommon to explicitly think of a probability distribution and instead use a method that captures some components (e.g., mean and variance) of a distribution. One difficulty in building matrix models is that even when we know the true distribution of stage duration, incorporating the distribution precisely might not be possible. Although matrix models can describe accurate dynamics when within‐stage age distributions are stable (Caswell, 2001), this assumption is not necessarily satisfied (Runge & Roff, 2000). More importantly, the effects of the distributions on population growth rate cannot be examined when specific distributions of interest cannot be expressed.

**Figure 1:**
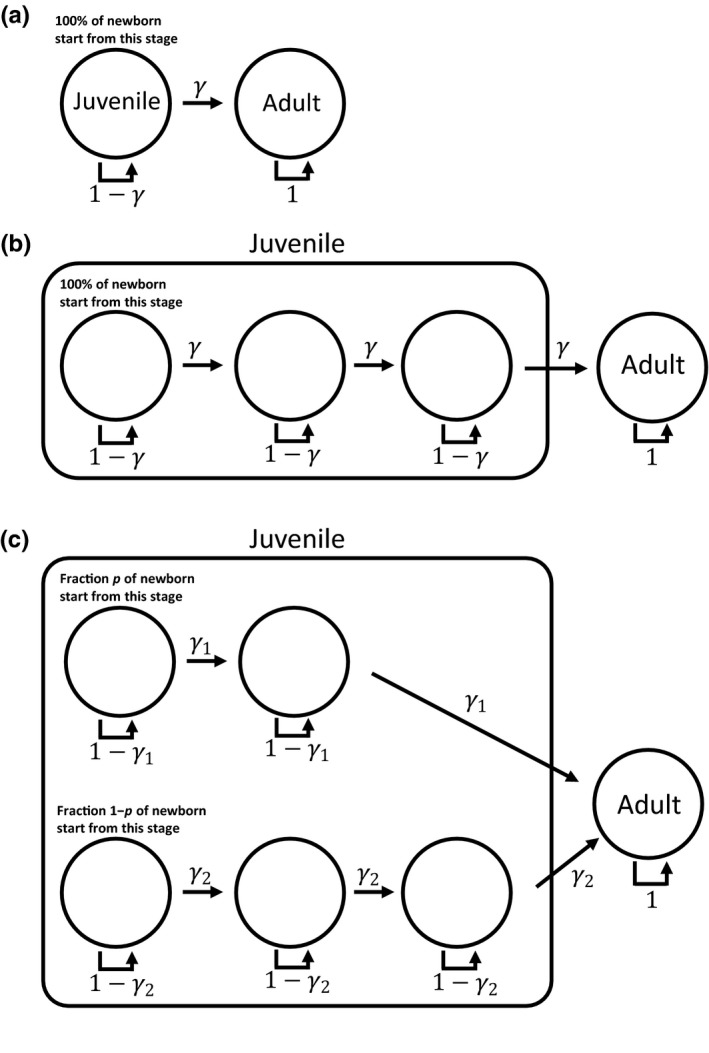
Diagrams describing stage transitions given that individuals survive. Juvenile duration follows a (a) geometric distribution, (b) negative binomial distribution, and (c) mixture of two negative binomial distributions. Each arrow indicates an event, and the associated value (e.g., γ) is the probability that the event takes place given that an individual survives

To illustrate the difficulty in modeling stage duration, a species consisting of two stages (juvenile and adult) is considered in which we are interested in modeling the distribution of juvenile duration. A standard method for describing stage duration assumes that a juvenile either leaves the juvenile stage (i.e., becomes an adult) with probability γ or remains as a juvenile with probability 1 − γ for a given time step if it survives (Figure 1a). In other words, the stage transition (per time step) is a Bernoulli process with success probability γ, with the stage duration of a juvenile realized by the number of Bernoulli trials required to have one success, which is known as a geometric distribution. A geometric distribution might be appropriate in some cases, but is highly restrictive. For example, the expected duration of juvenile stage in the whitefly *Bemisia argentifolii* is similar when they are raised on eggplant (17.31 days) and on tomato (17.96 days), but the associated variances are different: 44.47 on eggplant and 77.00 on tomato (Tsai & Wang, 1996). A geometric distribution whose mean is 17.5 must have its variance as 288.75, which suggests that it is inappropriate for both cases. Furthermore, as shown in the example, distributions can have the same mean while having different variances. Geometric distributions cannot have different variances when they have the same mean. As such, cases in which the use of a geometric distribution is appropriate are limited.

Extensions of the geometric distribution are used to describe stage duration more flexibly. A natural extension is the sum of geometric distributions known as the negative binomial distribution (Caswell, 2001). A negative binomial distribution can be interpreted as the number of Bernoulli trials required to have *k* successes. Figure 1b shows an example with *k* = 3. To become an adult, a newly born juvenile must go through identical Bernoulli trials until three successes are achieved. In this example, the juvenile stage contains three stages known as pseudostages. Pseudostages are created for convenience (e.g., simply to make *k* > 1) and typically do not represent biologically meaningful stages such as age. Ages are implicit in the models considered in this study, but matrix models that consider both age and stage explicitly have also been developed (Caswell, 2012; Roth & Caswell, 2016, 2018).

Mixtures of two negative binomial distributions have also been suggested to create even more flexible distributions (Birt et al., 2009). In the example shown in Figure 1c, there are two independent negative binomial distributions describing the duration of the juvenile stage, and each juvenile follows one of the two distributions for which the probabilities that a newborn enters the first and second negative binomial distributions are *p* and 1 − *p*, respectively. Each negative binomial distribution is characterized by two parameters (*k*_1_,$\gamma_{1}$) and (*k*_2_,$\gamma_{2}$). Having five parameters (*p*,* k*_1_, $\gamma_{1}$, *k*_2_, and $\gamma_{2}$), mixtures of negative binomial distributions are the most flexible among the distributions described here. In fact, the geometric distribution is a special case of the negative binomial distribution (i.e., *k* = 1) and the negative binomial distribution is a special case of the mixture distribution (i.e., *p* = 1 or *p* = 0). In this study, mixtures of two negative binomial distributions are referred to as a mixture distribution unless otherwise stated.

Although a mixture distribution is more flexible than geometric and negative binomial distributions, actual distributions of stage duration might differ significantly from it. Therefore, it is important to know how well a mixture distribution can approximate other potential duration distributions. A previous study found that a mixture distribution cannot approximate common distributions such as gamma and lognormal distributions effectively when the parameters of the mixture distribution are estimated heuristically (Lee & Okuyama, 2017). However, the fact that a mixture distribution does not perform well with heuristic parameter estimation (described in Appendix) does not necessarily indicate the failure of the mixture distribution when the parameters are estimated differently. The heuristic method is similar to the method of moments, which uses only information contained in moments (e.g., the mean and variance), and it might make little sense when the assumed distribution is known to be wrong (e.g., using a mixture distribution to approximate a gamma distribution), and when information not contained in moments influences population dynamics. Maximum‐likelihood estimation accounts for other properties of distributions through a fuller utilization of data (e.g., not just the mean and variance). This study examined the performance of a mixture distribution in approximating other target distributions when model parameters were estimated using a maximum‐likelihood method.

## 2 METHODS

### 2.1 Matrix population model

A species that experiences two life stages, such as the one shown in Figure 1, is considered. The matrix model uses a postbreeding census formulation (Case, 2000; Caswell, 2001) to keep track of the number of individuals in each stage and assumes that the duration of juvenile stage *T*_*J*_ follows a mixture distribution. For example, Figure 1c describes the stage transitions when *k*_1_ = 2 and *k*_2_ = 3. Additional parameters describing survival and reproduction must be specified to complete a full demographic model. $\sigma_{J}$ and $\sigma_{A}$ are the survival probabilities for juvenile and adult, respectively, and *m* is the expected number of female offspring produced by an adult female. All parameters describe demographic processes that take place in one discrete time step (e.g., day, week, or year), and an appropriate time step should depend on organisms (Cull, 1980). The model assumes that males do not limit reproduction and keeps track only of females (Caswell, 2001).

Matrix population models can be described as

(1) N(t+1)=AN(t)

where **N**(*t*) is a vector that consists of the number of individuals in each stage at time *t*. For example, for Figure 1c, **N**(*t*) is a vector length of six (i.e., five pseudostages and one adult stage). **A** completely summarizes the demographic processes. Using the postbreeding census method, a mixture distribution‐based model corresponding with Figure 1c is,

(2) $$A\;=\;\left(\begin{array}{ccccccccccc} P_{1} & & pG_{1}b & & 0 & & 0 & & pG_{2}m & & p\sigma_{A}m\\ G_{1} & & P_{1} & & 0 & & 0 & & 0 & & 0\\ 0 & & \left(1-p\right)G_{1}m & & P_{2} & & 0 & & \left(1-p\right)G_{2}m & & \left(1-p\right)\sigma_{A}m\\ 0 & & 0 & & G_{2} & & P_{2} & & 0 & & 0\\ 0 & & 0 & & 0 & & G_{2} & & P_{2} & & 0\\ 0 & & G_{1} & & 0 & & 0 & & G_{2} & & \sigma_{A} \end{array}\right)$$

where $P_{i}\;=\;\sigma_{J}\left(1-\gamma_{i}\right)$ is the probability that a juvenile survives and remains in the same pseudostage, with i∈{1,2} representing one of the two negative binomial distributions. $G_{i}\;=\;\sigma_{J}\gamma_{i}$ is the probability that a juvenile in a pseudostage survives and advances to the next stage (another pseudostage or the adult stage). The model assumes that the stage duration *T*_*J*_ is a latent trait (i.e., determined at the birth), and an observed distribution can significantly differ from the distribution of *T*_*J*_ because some individuals die before becoming adults (further discussed below, also see Ergon, Yoccoz, & Nichols, 2009). The distribution of latent stage duration coincides with the observed distribution only when all individuals survive till adults (i.e., $\sigma_{J}\;=\;1$).

### 2.2 Model parameters and analysis

Maximum‐likelihood estimates (MLEs) of (*p*,* k*_1_, $\gamma_{1}$, *k*_2_, and $\gamma_{2}$) were used to create matrix models. When *f* is the true distribution of stage durations (i.e., *T*_*J*_ ~ *f*), a matrix model uses a mixture distribution to approximate *f*. For a given true (or target) distribution of juvenile duration *f* (specific distributions will be described below), the maximum‐likelihood parameters of a mixture distribution were estimated from 1,000 random samples generated from *f*.

Once the MLEs are determined, a population matrix (e.g., Equation (2)) can be fully specified with the three additional parameters $\sigma_{J}$, $\sigma_{A}$, and *m*. Specific parameter values are discussed below. In this study, **A** is an irreducible primitive matrix that makes the population ergodic according to the Perron‐Frobenius theorem (Caswell, 2001). In other words, the population growth rate eventually converges to a fixed value regardless for any positive initial condition, and the asymptotic population growth rate (i.e., the finite rate of increase) is represented by the dominant eigenvalue of **A**. In one simulation run, random samples from *f* are used to parametrize **A**, and the finite rate of increase is estimated from **A**. Because the finite rate of increase fluctuates as a result of random sampling from *f*, the average from 100 simulation runs was used to represent the expected value of the finite rate of increase.

### 2.3 Individual‐based models (IBMs)

The matrix model described above assumes that juvenile duration follows a mixture distribution. When the true distribution, *f*, is not a mixture distribution, the matrix model is an approximation. Examining the performance of matrix models when *f* is not a mixture distribution requires knowing the true population growth under *f*. An IBM was created to obtain population growth for various instances of *f*. Because matrix models describe simple demographic processes that occur in discrete time steps, corresponding IBMs can be created by simulating the demographic processes. For example, the survival of individuals is simulated as a Bernoulli trial with the survival probabilities $\sigma_{J}$ (for juveniles) and $\sigma_{A}$ (for adults). For each newly born individual, the duration of its juvenile stage is simulated from *f*. If the stage duration of a juvenile is *x*, the juvenile becomes an adult if it survives *x* time steps. Each adult reproduces *m* offspring on average, and the number of offspring was simulated from a Poisson distribution with mean *m*.

The finite rate of increase of a population can be estimated by simulating the IBM for many time steps. In particular, *N*(*t *+* *1)/*N*(*t*) converges to the finite rate of increase, where *N*(*t*) is the number of individuals at time *t* (the sum of all individuals at time *t*). *N*(*t *+* *1)/*N*(*t*) will fluctuate some even after convergence as a result of the stochastic nature of the model. The geometric mean of *N*(*t *+* *1)/*N*(*t*) from the last 1,000 time steps of 2,000 total time steps was used to represent the finite rate of increase. It was confirmed that a burn‐in period of 1,000 was sufficient to obtain convergence.

### 2.4 Comparison of the matrix model and IBM results

Estimates of the finite rate of increase from the matrix model and the IBM were compared under various choices of *f* in *T*_*J*_ ~ *f*. The IBM describes true (target) dynamics, and thus, a difference in prediction between an IBM and the corresponding matrix model indicates an inaccuracy in the matrix model. Four parametric distributions were considered for *f*: (a) zero‐truncated Poisson distributions, (b) zero‐truncated over‐dispersed Poisson distributions [explained below], (c) (discrete) gamma distributions, and (d) (discrete) lognormal distributions. The zero‐truncated distributions were used because a juvenile duration of zero indicates that adults are directly producing adults, which was assumed to be impossible in this study.

Negative binomial distributions can be defined in multiple manners. One form of negative binomial distribution was already described above (e.g., Figure 1b; also see Appendix). Another formulation uses two parameters μ and *k*, where the mean is μ and the variance is μ + μ^2^/*k* (Bolker, 2008). This form of negative binomial distributions is referred as over‐dispersed Poisson distributions in this study to avoid confusion between the two negative binomial distributions.

For gamma and lognormal distributions, random samples were rounded up to the nearest integer (i.e. ceiling). Taking the ceiling eliminates zero and results in discrete random samples. Because matrix models are discrete time models, the realized stage durations must be discrete. As discussed above, a previous study found that mixture distributions (based on the heuristic method) fail to approximate gamma and lognormal distributions, and thus, these distributions present good tests for the current study. Furthermore, these distributions are among the most commonly used distributions for describing nonnegative random variables.

To examine the performance a mixture model in approximating *f* (i.e., *T*_*J*_ ~ *f*), the mean and variance of *f* were varied systematically when possible. To obtain a target distribution with specified mean *E*(*T*_*J*_) and variance Var(*T*_*J*_), the method of moment estimates was used (e.g., the shape and scale parameters of a gamma distribution, respectively, are *E*(*T*_*J*_)^2^/Var(*T*_*J*_) and Var(*T*_*J*_)/*E*(*T*_*J*_)). For gamma and lognormal distributions, *E*(*T*_*J*_) and Var(*T*_*J*_) are the mean and variance without the ceiling, and thus, the actual mean and variance differ slightly from *E*(*T*_*J*_) and Var(*T*_*J*_). For zero‐truncated Poisson distributions, only the mean was set to a desired value. Zero‐truncated Poisson distributions have one parameter λ with *E*(*T*_*J*_) = $\lambda /(1-e^{-\lambda })$, and Var(*T*_*J*_) = *E*(*T*_*J*_)(1 +  λ − *E*(*T*_*J*_)). Therefore, setting the expected duration *E*(*T*_*J*_) automatically determines the associated variance.

For this study, producing meaningful comparisons requires that the IBM and the matrix model must be defined consistently. In other words, if a mixture distribution is used as *f* in an IBM, then the IBM and the corresponding matrix model must produce the same finite rate of increase. This check is shown in *Results*. In addition, the performance of matrix models with the heuristic method is also included as a reference. For convenience, matrix models parametrized by the heuristic and likelihood methods, respectively, are referred to as the heuristic and likelihood models.

Parameters associated with a true scenario are the parameters of *f* (e.g., mean and variance of juvenile duration), $\sigma_{J}$, $\sigma_{A}$, and *m*. Because the theoretically possible parameter space is infinitely large, a specific parameter space must be specified. Parameter values were informed from the life cycle of the oriental fruit fly (*Bactrocera dorsalis*) (Fang et al., 2011). The matrix **A** in Equation (1) describes demographic processes that take place in one day. The sum of the average durations of juvenile stages is approximately 30 days, and the associated variance is approximately three at 25°C. To reflect this, the average juvenile duration *E*(*T*_*J*_) was varied from three to 35 days in this study, and the ratio of variance to mean Var(*T*_*J*_)/*E*(*T*_*J*_) was varied from 0.1 to 2.0. The probability that a newly born juvenile survives to become an adult *s*_*J*_ was varied from 0.05 to 0.95 to consider as nearly full a parameter range as possible. The daily juvenile survival probability $\sigma_{J}$ was computed from the relationship, $\sigma_{J}^{E(T_{J})}\;=\;s_{J}$. The daily adult survival probability $\sigma_{A}$ was varied from 0.5 to 0.98, corresponding to the average adult duration (i.e., adult longevity) from two to 50 days, whereas an adult *B. dorsalis* survives approximately 40 days under laboratory conditions. The median of daily fecundity is approximately 15 eggs. Assuming a 1:1 sex ratio, the number of female eggs is approximately seven. To reflect this, *m* was varied from five to 15. Thus, the parameter space was set liberally to reflect a much greater parameter space than might be realized by *B. dorsalis*. Furthermore, as will be explained below, although $\sigma_{J}$, $\sigma_{A}$, and *m* influence the finite rate of increase, they do not qualitatively influence how the distribution of duration influences the finite rate of increase. Therefore, the survival and fecundity parameters are not crucial factors in this study. The source code in R used in the analysis is provided as Supporting Information.

## 3 RESULTS

**Figure 2:**
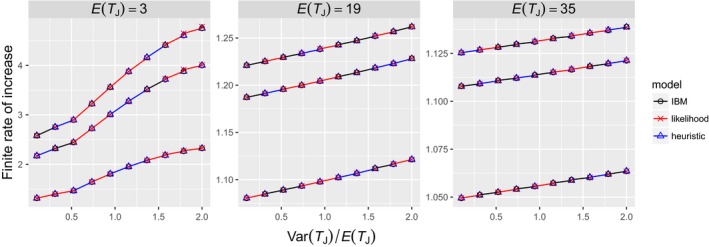
Relationship between the distribution of juvenile duration and the finite rate of increase. The true distribution used in the IBM is a mixture distribution whose parameters were determined using the heuristic method. *E*(*T*_*J*_) and Var(*T*_*J*_) were systematically varied. For example, when Var(*T*_*J*_)/*E*(*T*_*J*_) = 2 and *E*(*T*_*J*_) = 3, Var(*T*_*J*_) = 6. In each *E*(*T*_*J*_) figure, it appears that there are three lines corresponding with *s*_*J*_ = 0.95 (top line), *s*_*J*_ = 0.5 (middle line), and *s*_*J*_ = 0.05 (bottom line) where $\sigma_{J}^{E(T_{J})}\;=\;s_{J}$, but each of the three lines consists of three additional overlapping lines, because the three models show identical results. $\sigma_{A}\;=\;0.95$, *m* = 10

When the distribution of juvenile duration in an IBM, *f*, is a mixture distribution whose parameters are determined by the heuristic method (Appendix), the IBM and matrix models result in identical population growth rates for all parameter combinations (Figure 2), showing that the IBM and the matrix models are defined consistently such that comparisons based on other distributions of *f* are meaningful. In addition, the parameters of mixture distributions in the matrix models were estimated from random samples of *f* rather than through using the known parameters of *f*, indicating that the parameter estimation methods performed sufficiently well.

**Figure 3:**
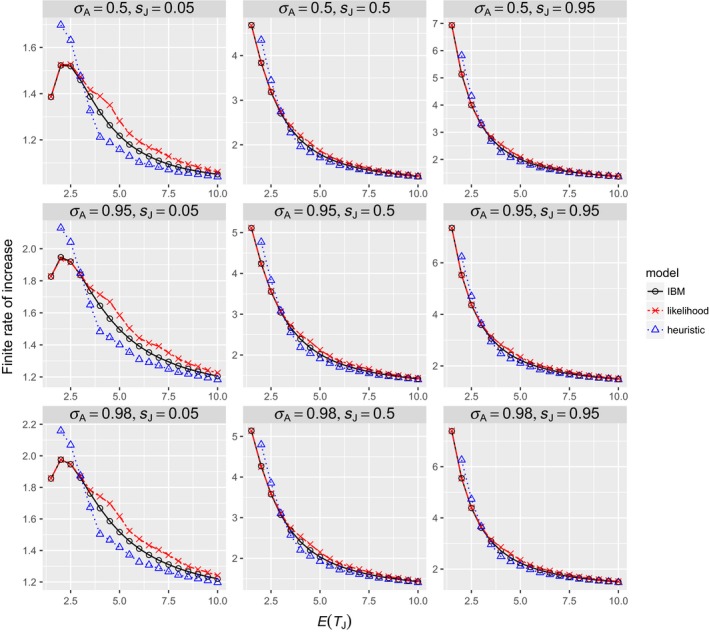
Relationship between the distribution of juvenile duration and the finite rate of increase. The true distribution used in the IBM is a zero‐truncated Poisson distribution with mean *E*(*T*_*J*_). *m* = 10

Both the heuristic and likelihood models performed generally well when *f* is a zero‐truncated Poisson distribution (Figure 3). Figure 3 focuses on short juvenile durations (*E*(*T*_*J*_) ≤ 10), because the difference among the models becomes smaller as *E*(*T*_*J*_) becomes longer, as can also be inferred from the figure. When *E*(*T*_*J*_) is greater than 3 days, the likelihood model overestimates population growth rates, whereas the heuristic model underestimates them.

Among offspring that are born at the same time, those with short juvenile durations contribute to the population growth disproportionately more than those with longer juvenile durations. For example, if we compare a juvenile with a duration of 1 day and another with a duration of 5 days, the former juvenile will start producing offspring at the next breeding event, and there will already be grandchildren in the following time step even when the latter individual is still juvenile. Consequently, producing two offspring whose juvenile durations are 1 and 5 days, respectively, can result in a greater population growth rate than producing two offspring whose juvenile durations are both 3 days, even though the average juvenile duration is the same for both cases when not considering survival. This is a positive effect of Var(*T*_*J*_). Depending on the strength of the positive effect of Var(*T*_*J*_) and the negative effect of *E*(*T*_*J*_), the relationship between the population growth rate and *E*(*T*_*J*_) is not monotonic (e.g., when *s*_*J*_ = 0.05 and *E*(*T*_*J*_) < 3). The likelihood model closely captured this pattern, but the heuristic model failed. In addition, there are no estimates from the heuristic model when *E*(*T*_*J*_) = 1.5 because it produces infeasible parameters.

**Figure 4:**
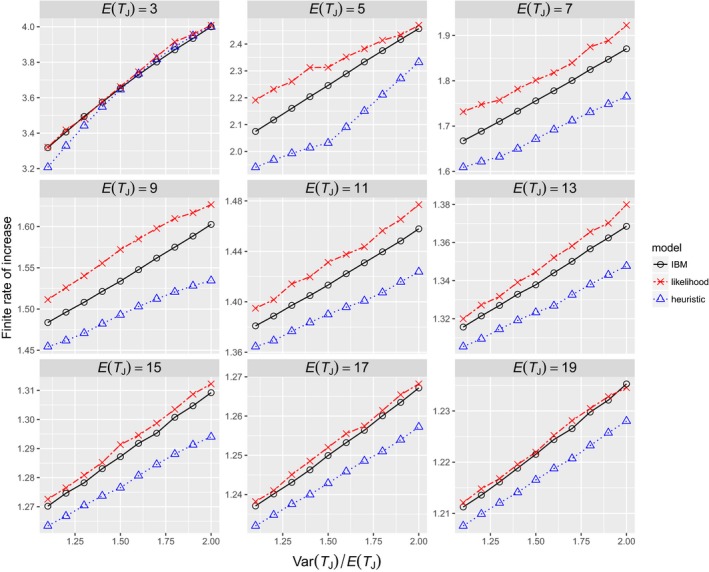
Relationship between the distribution of juvenile duration and the finite rate of increase. The true distribution used in the IBM is a zero‐truncated over‐dispersed Poisson distribution with mean *E*(*T*_*J*_) and variance Var(*T*_*J*_). *m* = 10, $\sigma_{A}$ = 0.95, *s*_*J*_ = 0.5

When the true distribution *f* is a zero‐truncated over‐dispersed Poisson distribution, the likelihood model overestimates and the heuristic model underestimates the population growth rate (Figure 4). In Figure 4, results only from Var(*T*_*J*_)/*E*(*T*_*J*_) > 1.1 are shown because the variance of an over‐dispersed Poisson distribution must be greater than its mean. For a given mean duration *E*(*T*_*J*_), the variance does not have a strong effect on the bias, but the mean duration strongly influences the bias. For example, the likelihood model performs relatively poorly when *E*(*T*_*J*_) takes on some intermediate values, but when *E*(*T*_*J*_) is very low or high, the bias becomes negligible. On the other hand, estimates from the heuristic model are significantly lower than the true values regardless of *E*(*T*_*J*_).

**Figure 5:**
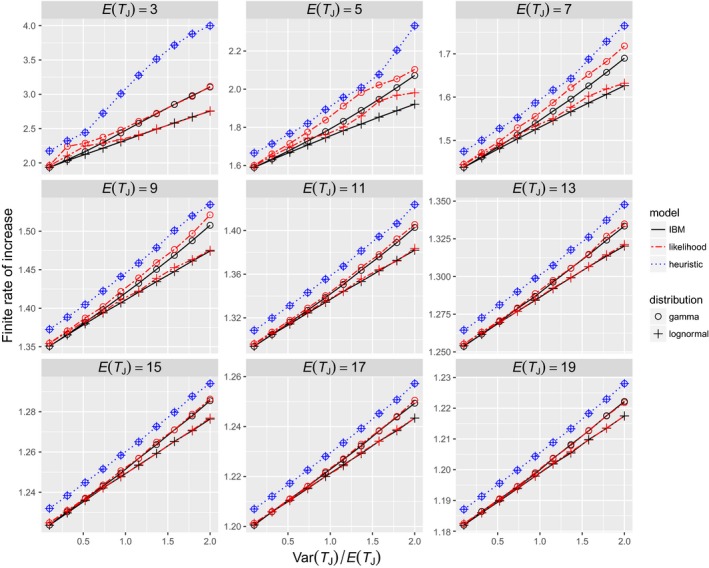
Relationship between the distribution of juvenile durations and the finite rate of increase. The true juvenile durations are the ceilings of numbers from a gamma or lognormal distribution with mean *E*(*T*_*J*_) and variance Var(*T*_*J*_). *m* = 10, $\sigma_{A}$ = 0.95, *s*_*J*_ = 0.5

For a given combination of mean and variance, the gamma distribution results in a higher population growth rate than the lognormal distribution (Figure 5). The heuristic model could not describe the difference between the two distributions because its parameters are completely determined by the mean and variance of *f*. In contrast, the likelihood model predicted the difference. Both models generally overestimate population growth rates, but the biases are much greater for the heuristic model than for the likelihood model. When *E*(*T*_*J*_) ≥ 13, the qualitative relationship between Var(*T*_*J*_)/*E*(*T*_*J*_) and the finite rate of increase does not change (i.e., the proportional biases do not change), even though the finite rate of increase decreases as *E*(*T*_*J*_) increases. For this reason, results from *E*(*T*_*J*_) > 19 are not shown in Figure 5.

**Figure 6:**
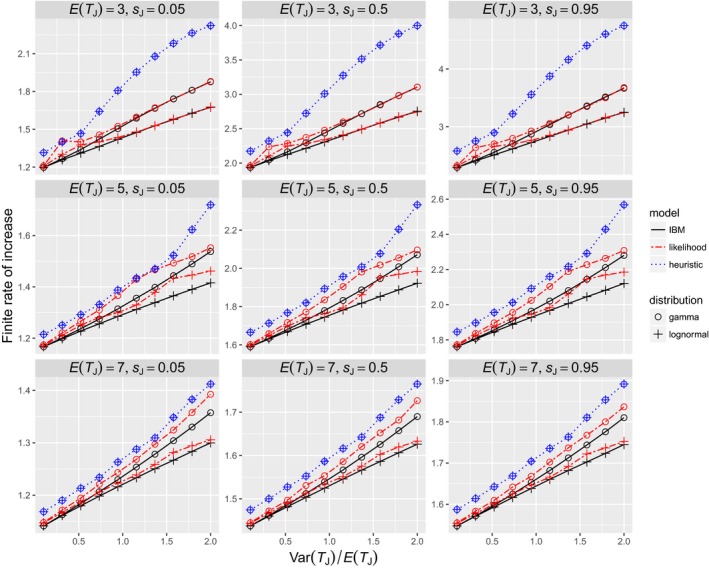
Relationship between the distribution of juvenile durations and the finite rate of increase. The true juvenile durations are the ceilings of numbers from a gamma or lognormal distribution with mean *E*(*T*_*J*_) and variance Var(*T*_*J*_). *m* = 10 and $\sigma_{A}\;=\;0.95$

The other parameters (i.e., *s*_*J*_, $\sigma_{A}$, and *m*) positively influence the finite rate of increase, but they do not qualitatively affect the relationship between Var(*T*_*J*_)/*E*(*T*_*J*_) and the finite rate of increase. To illustrate this point, the effect of *s*_*J*_ (where $\sigma_{J}^{E(T_{J})}\;=\;s_{J}$) is shown in Figure 6. For a given *E*(*T*_*J*_), changing *s*_*J*_ from 0.05 to 0.95 has little influence on the relationship other than that population growth rates generally increase with *s*_*J*_. The same is true for $\sigma_{A}$ and *m*.

## 4 DISCUSSION

Stage‐specific characteristics, such as the distribution of stage durations, influence the growth rate of a stage‐structured population. Because a probability distribution cannot be described fully in terms of its mean and variance, a heuristic model that depends solely on the mean and variance of a distribution will likely produce misleading conclusions. On the other hand, a likelihood model can track other properties of a distribution and reasonably approximates population growth rates under commonly used distributions, such as lognormal, gamma, and zero‐truncated (over‐dispersed) Poisson distributions.

It should be noted that the distribution of stage durations is defined for all individuals including those that do not reach the next stage. Suppose that *T* (the subscript *J* is omitted to indicate an arbitrary stage) follows a gamma distribution. Observed stage durations based on surviving individuals do not follow the gamma distribution unless the mortality (1‐σ) is negligible. In other words, when *Q* is the stage duration of individuals that survive to the next stage, *Q* = *T* only when σ=1. Because in a study, we only have data from *Q*, the effect of mortality on stage durations must be explicitly incorporated into the parameter estimation procedure. The probability that an individual survives *x* time steps and reaches the next stage is

(3) $$\text{Prob}\left(Q\;=\;x\right)\;=\;g\left(x\right)\sigma^{x}$$

where *g* is the probability mass function (e.g., a mixture of two negative binomial distributions) that is used in a matrix model. Supposing that there are *N* individuals initially, and that *S* individuals survive to the next stage, this process can be described as

(4) $$S∼\text{Binomial}(N,\sum_{x=1}^{\infty }g\left(x\right)\sigma^{x})$$

where the second argument of the binomial distribution is the probability parameter that describes the probability that an individual survives the focal stage. The maximum‐likelihood parameters of both *g* and σ can be obtained from these relationships. Ergon et al. (2009) provide a method for estimating the latent distribution *T* from capture–recapture data. When a matrix model is defined based on a latent distribution (e.g., Equation (2)), the estimation of the latent distribution is essential.

Relatively poor performance of the likelihood model when *E*(*T*_*J*_) is short is a valid concern, because none of the parametric distributions considered in this study is unrealistic. Although this study combined all sexually immature stages (e.g., egg and larva stages) into one stage, stage‐structured models may consider these stages explicitly (e.g., Bommarco, 2001; Lončarić & Hackenberger, 2013), making the duration of each stage short. Even when there are no distinct life stages such as larval and pupal stages, size‐dependent mortality is commonly reported (e.g., Grutter et al., 2017; Remmel & Tammaru, 2009). To capture size‐dependent mortality rates, a stage (e.g., juvenile stage) may be further subdivided into size classes (e.g., Crouse, Crowder, & Caswell, 1987), which also makes the duration of each class short. One way to improve approximation is to extend the mixture distribution. For example, mixtures of more than two distributions can be considered. It is useful to recognize that when there are *n* distinct stage durations (e.g., *n* = 4 when observed durations are always between 5 and 8 days), a multinomial distribution with probability parameters matching the proportions of observed durations can be considered a full model. Because a multinomial distribution with *n* possible outcomes can be expressed as a mixture of *n* constants (e.g., Figure 1c describes a binomial distribution with two possible outcomes (i.e., two or three) when $\gamma_{1}\;=\;\gamma_{2}\;=\;1$), mixtures of sufficiently many distributions will describe any observed data accurately. One can conduct model selection against the full model to determine the complexity of the required model.

When modeling a distribution, using a commonly used model (including the mixture distribution) for convenience might not be well advised. A distribution can be customized when information regarding it is available (e.g., the multinomial distributions discussed above). For example, there may be a minimal duration required for some stages (e.g., Dzierzbicka‐Głowacka, 2004; Oyarzun & Strathmann, 2011). If a model predicts some individuals stay only 2 days in a stage although at least 3 days are needed to develop into the next stage due to some biological constraints, this inaccuracy can be a cause of important mismatch between model predictions and observations. In a situation like this, adding a constant can assure the required duration in the stage and may describe the target distribution reasonably well. For example, even though the geometric distribution is very restrictive as discussed above, the sum of a constant and a geometric distribution is more flexible and can be expressed in matrix models. Although it is currently customary to report only the mean and variance of data, more detailed examination of stage duration would assist us in identifying important properties of duration distributions beyond the mean and variance, as well as appropriate models for approximating target distributions in matrix models.

## CONFLICT OF INTEREST

None declared.

## AUTHOR CONTRIBUTION

TO performed all work presented in this study.

## DATA ACCESSIBILITY

The computer code in R used in the analysis is available in Supporting Information.

## APPENDIX 1

### 1.1

This section describes the mixture distribution and the heuristic parameter estimation method discussed in the main text. When *T* is a random variable that follows a mixture distribution,

(5) $$T\;=\;pY_{1}\;+\;\left(1-p\right)Y_{2}$$

where *Y*_1_ and *Y*_2_ are random variables that follow independent negative binomial distributions such that *Y*_1_ ~ NegBin(*k*_1_,$\gamma_{1}$) and *Y*_2_ ~ NegBin(*k*_2_,$\gamma_{2}$) in which the probability mass function of NegBin(*k*,γ) is

(6) $$P\left(Y\;=\;y\right)\;=\;\left(\begin{array}{c} y-1\\ k-1 \end{array}\right)\gamma^{k}{\left(1-\gamma \right)}^{y-k}$$

that can be interpreted as the sum of *k* geometric distributions with parameter γ. p∈[0,1] is the mixture probability such that *T* is a single negative binomial distribution when *p* = 0 or *p* = 1. The mean and variance of the mixture distribution (Equation (5)), respectively, are

(7) $$E\left(T\right)\;=\;pE\left(Y_{1}\right)\;+\;\left(1-p\right)E\left(Y_{2}\right)$$

(8) $$\text{Var}\left(T\right)\;=\;p\left(E{\left(Y_{1}\right)}^{2}\;+\;\text{Var}\left(Y_{1}\right)\right)\;+\;\left(1-p\right)\left(E{\left(Y_{2}\right)}^{2}\;+\;\text{Var}\left(Y_{2}\right)\right)-E{\left(T\right)}^{2}$$

where *E*(*Y*_*i*_) = *k*_*i*_/$\gamma_{i}$ and Var(*Y*_*i*_) = *k*_*i*_(1‐$\gamma_{i}$)/$\gamma_{i}^{2}$ (i∈{1,2}).

The heuristic method determines the parameters as follows. A mixture distribution with mean *E*(*T*) and Var(*T*) has

(9) $$k_{1}\;=\;\left\lfloor \frac{E{\left(T\right)}^{2}}{\text{Var(T) + E(T)}}\right\rfloor$$

(10) $$k_{2}\;=\;\left\lceil \frac{E{\left(T\right)}^{2}}{\text{Var}(T)\;+\;E(T)}\right\rceil$$

where ⌊x⌋ and ⌈x⌉ are the floor and ceiling of *x*, respectively. The method further assumes that $\gamma \;=\;\gamma_{1}\;=\;\gamma_{2}$, leaving two remaining free parameters (*p* and γ) that can be determined by solving Equations (7) and (8) (i.e., two equations and two unknowns). When the data are available, *E*(*T*) and Var(*T*) are replaced by the sample mean and variance.
